# Aesthetics Are in the Eye of the Beholder: Evaluation of the Nasolabial Appearance After Primary Cleft Lip Repair

**DOI:** 10.3390/jcm14217501

**Published:** 2025-10-23

**Authors:** Michala Ivanic-Sefcikova, Vasco Starke, Marc Brommer, Lukas Groessing, Wolfgang Zemann, Carolin Bestendonk, Michael Schwaiger

**Affiliations:** 1 Department of Oral and Maxillofacial Surgery, Medical University of Graz, Auenbruggerplatz 5, 8036 Graz, Austria; michala.sefcikova@medunigraz.at (M.I.-S.); marc.brommer@stud.medunigraz.at (M.B.); lukas.groessing@medunigraz.at (L.G.); wolfgang.zemann@medunigraz.at (W.Z.); michael.schwaiger@medunigraz.at (M.S.); 2 Department of Oral and Maxillofacial Surgery, Charité—Universitätsmedizin Berlin, Augustenburgerplatz 1, 13353 Berlin, Germany; carolin.bestendonk@charite.de

**Keywords:** Cleft Lip Surgery, postoperative outcome, primary cleft lip revision, SymNose, cleft aesthetic rating scale, nasolabial appearance

## Abstract

**Background/Objectives**: Primary cleft lip repair is a procedure to restore lip function and harmonious nasolabial appearance. However, the actual effects on nasolabial appearance have been insufficiently investigated. The aim of this study was therefore to critically review surgical results using well-validated objective and subjective methods and standardised outcome measures. **Methods**: A total of 26 non-syndromic patients with unilateral cleft lip +/− cleft palate were assessed retrospectively. Uniform 2D photographs of the nasolabial region were objectively analysed using SymNose software. The Cleft Aesthetic Rating Scale was employed for the subjective evaluation by a total of 255 observers with different backgrounds and experience levels in cleft surgery. **Results**: Significant positive correlation was identified between objective measurement of upper lip asymmetry and subjective assessment (*p* = 0.02). Furthermore, a significant difference emerged in the assessment of the nasolabial region depending on the rater’s professional background (*p* < 0.001). A major discrepancy was observed between the rating of maxillofacial consultants and those of laypersons and patients affected by cleft lip +/− cleft palate. **Conclusions**: The evaluation of attractiveness depends significantly on the professional background of the observer. The more interaction people have with individuals after cleft lip repair, the less they consider the aesthetics conspicuous. Achieving the highest possible postoperative nasolabial symmetry should be a main objective in primary cleft lip repair.

## 1. Introduction

Cleft lip, with or without cleft palate, is the most common congenital malformation, with an overall prevalence of 7.94 per 10,000 births, varying in frequency depending on ethnic group and gender [1]. Primary cleft lip repair is generally performed in the first months of life, and multiple surgical techniques for unilateral cleft lip repair have been described in the literature [2,3,4,5,6]. The main goal of these procedures, in addition to functionality, is to achieve a harmonious nasolabial appearance. This includes restoring lip function (by re-suturing the orbicularis oris muscle) and creating a balanced upper lip, with appropriate philtrum length, a defined vermilion border, harmonious volume distribution in the upper lip vermilion, an inconspicuous lip scar, and a stable result over time. The approach of “do not touch the nose in the primary cleft lip repair”, with surgical intervention focused strictly on the lip tissue, was abandoned. To achieve an optimal aesthetic result, including proper 3D positioning of the ala and satisfactory nostril form, the cleft lip repair should be combined with primary septorhinoplasty [7]. However, residual cleft-related features may persist after primary correction and may continue to considerably impair the patient’s nasolabial appearance. As these features can compromise quality of life during adolescence and adulthood, initial treatment and follow-up care should be carried out as meticulously as possible to allow for timely planning of secondary procedures, if desired by the patient.

In the literature, many studies have attempted to objectively assess the outcomes of primary cleft lip repair techniques, with a focus on evaluating symmetry as the criterion for success. The majority of these studies rely on quantitative measurements obtained from 2D photographs [8,9]. Nonetheless, in recent years, 3D stereophotogrammetry has gained increasing popularity [10]. Other studies categorise postoperative outcomes based on qualitative assessments, using reference photographs to illustrate a range of results [11,12]. In such procedures, respondents classify the rated images by comparing them to standardised reference photographs, with the aim of enhancing the objectivity and consistency of aesthetic ratings. However, these methods of assessment fail to address the core issue—the actual impact on subjectively perceived facial aesthetics.

It is undeniable that people with faces perceived as attractive are treated more positively [13]. Attractiveness is a subjective phenomenon, but there are attempts to define its objective biomedical or anatomical parameters. Symmetry is considered one of these parameters [14]. However, it remains highly uncertain whether greater symmetry in the nasolabial region of patients with corrected cleft lip, as quantified by objective metrics, necessarily corresponds to enhanced perceptions of aesthetic harmony and attractiveness. Moreover, the literature offers little clarity on how professional background and personal experience with patients affected by cleft lip with or without cleft palate influence the assessment of aesthetic outcomes [15].

This study aims to focus on these overlooked factors and fill this literature gap. The primary objective of this study was therefore to determine the extent to which subjective aesthetic assessments align with objectively measured nasolabial symmetry and to evaluate whether professional background and familiarity with individuals affected by cleft lip influence perceptions of facial attractiveness. This paper intends to address the following question: Are aesthetics in the eye of the beholder regarding the appearance and specific features of cleft lip?

## 2. Materials and Methods

### 2.1. Study Population

**Figure 1:**
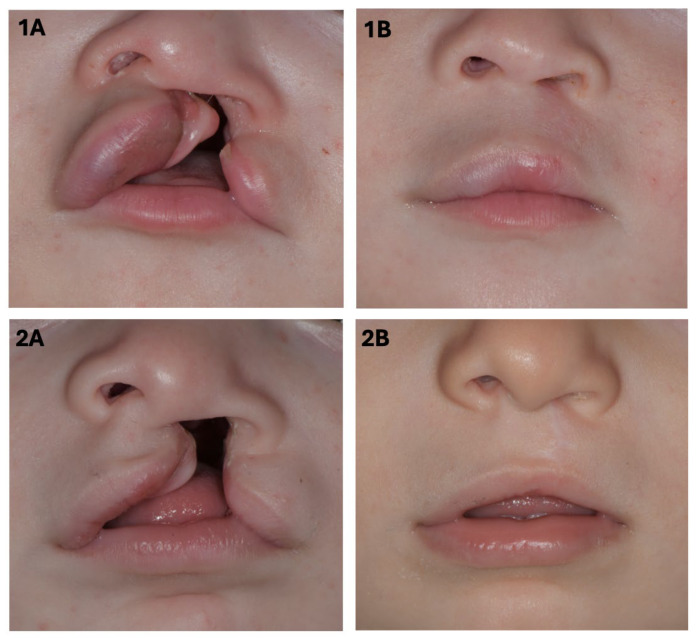
Images pre- (**1A**,**2A**) and post- (**1B**,**2B**) surgery of two patients with complete unilateral cleft lip, jaw, and palate, who underwent cleft lip repair using Fisher’s anatomical subunit approximation technique.

This retrospective study included Caucasian patients with non-syndromic unilateral cleft lip +/− palate (UCL(P)) who had received primary cleft lip repair at the age of 3 to 6 months between May 2019 and February 2024 at the Department for Oral and Maxillofacial Surgery at the Medical University of Graz. Cleft lip repair was performed using the anatomical subunit approximation technique described by D.M. Fisher (Figure 1) [6]. In all cases, the procedure was carried out by the same experienced senior surgeon (M.S.).

The inclusion criteria were the above-mentioned diagnosis and the availability of sufficient postoperative photographic documentation obtained between 6 and 36 months after surgery. Participants who did not meet these criteria were excluded. Ethical approval was obtained from the local ethics committee (EK number: 35-490 ex 22/23; Medical University of Graz) and written informed consent was obtained from the legal guardians of the minors before inclusion in the study.

### 2.2. Objective Evaluation

**Figure 2:**
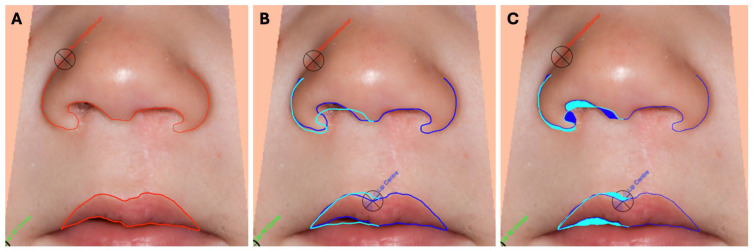
(**A**): Manual placing of the tracing points and contouring of the lower edge of the nose and upper lip. (**B**,**C**): SymNose analysis of the asymmetry of the nasal base, the asymmetry of the upper lip, and the asymmetry of the upper lip in relation to the midline of the face and to assess the percentage mismatch. ((**B**): mismatch display in lines; (**C**): mismatch display in areas).

To objectively evaluate the surgical outcome in terms of nasolabial symmetry, 26 standardised and anonymised 2D patient photographs (frontal view) were analysed using SymNose software (Version 8.1; Brian Pigott 2007–2017). This scientifically validated programme was developed to quantitatively measure facial symmetry in patients with cleft lip and palate [8,9,16]. Detailed descriptions of the software and its application are available in the literature [8,16]. The following three parameters were selected for this study: (1) nasal base asymmetry (CAFR); (2) upper lip asymmetry (CA lip); and (3) upper lip asymmetry in relation to the facial midline (UA lip). Figure 2 illustrates the technical procedure (Figure 2). Two examiners (described as rater 1 and rater 2) assessed the photographs independently on two different occasions (described as exam 1 and 2) to avoid bias.

### 2.3. Subjective Evaluation

The postoperative outcome was retrospectively evaluated on a subjective level by raters with different personal and professional backgrounds who appraised the nasolabial appearance of the patients. The rating participants included laypersons, cranio-maxillofacial surgeons (CMFS) with varying levels of experience in cleft surgery (residents and consultants), patients with cleft condition, as well as direct relatives of cleft-affected individuals. None of the relatives were associated with the patients whose nasolabial region was depicted in the photographs undergoing assessment. The laypersons were recruited from a diverse cross-section of society with no particular connection to cleft surgery through direct personal recruitment.

**Figure 3:**
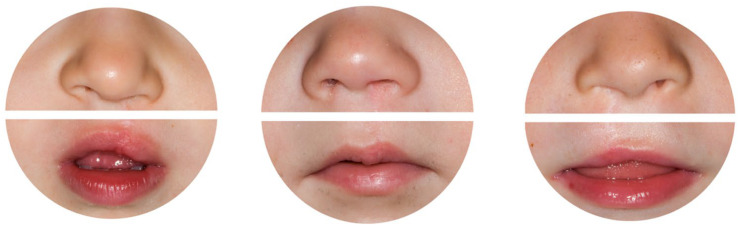
Standardised 2D photographs used for subjective assessment are depicted as an example.

The validated 5-point Cleft Aesthetic Rating Scale (CARS) was used to rate nasolabial appearance [12,17]. Using this comprehensive ranking method, the same standardised postoperative photographs, as previously measured objectively, were evaluated from A/1 (very good) to E/5 (very poor) (Figure 3). The following nasolabial features were part of the evaluation: nose tip symmetry, nostril flaring, lip symmetry, lip height, and vermillion border.

### 2.4. Statistical Analysis

Descriptive analyses, including mean, range, frequency, and standard deviation (SD), were performed. Regarding comparative analysis, a *T*-test and ANOVA (analysis of variance) followed by a post hoc test were used to determine differences in the rating of the rater subgroups. The Pearson correlation coefficient was used to assess the correlations between the objective and subjective assessments. The significance level for all analyses was set at 5% (*p* < 0.05). Statistical calculations were conducted using SPSS Statistics (IBM SPSS Statistics for Windows, Version 26.0. Armonk, NY, USA: IBM).

## 3. Results

This study included a total of 26 patients (13 males and 13 females) with a mean age of 21.42 months (±11.52). The mean follow-up was 15.23 months (±10.47). The left side was affected in 21 cases, the right side in 5 cases.

### 3.1. Objective Assessment

Both intra- and interrater reliability demonstrated very strong consistency across all three parameters measured (Table 1). The CAFR parameter demonstrated the lowest degree of asymmetry, with an average value of 19.94% (range: 7.86–37.77; SD ± 7.09), followed by 22.09% (range: 8.49–51.87; SD ± 9.45) for the UA lip and 22.43% (range: 8.38–44.28; SD ± 9.2) for the CA lip, respectively (Table 2). The consolidated data from rater 1 and 2 and exam 1 and 2 were utilised for further analysis.

### 3.2. Subjective Assessment

A total of 255 people with different professional and personal backgrounds were recruited for the subjective evaluation. The majority of assessors (*n* = 167) were laypersons. A total of 32 patients with cleft lip +/− palate were included and 38 relatives of these patients. Additionally, there were nine residents in CMFS and an equal number of CMFS consultants.

ANOVA revealed a statistically significant difference in the ratings of respondents with respect to their backgrounds (*p* < 0.001). Regarding labial aesthetics, laypersons were the most critical, assigning the highest scores (mean = 2.69; SD ± 0.44), closely followed by the group of patients with cleft condition (mean = 2.68; SD ± 0.70). Concerning nasal appearance, the patient group was the most discerning, with an average score of 2.51 ± 0.68, followed by the layperson group (mean = 2.40; SD ± 0.38). In contrast, the professional subgroups, CMFS residents and consultants, were the most optimistic in their assessment of the nasolabial region (Table 3).

### 3.3. ANOVA

To compare the subgroup ratings separately and scrutinise the significant differences between them, a post hoc test was utilised. The most notable finding was the significant difference in evaluation of lip region between laypersons and CMFS consultants (*p* < 0.001), followed by significant differences when compared to CMFS residents (*p* = 0.019) and patient’s relatives (*p* = 0.038). Furthermore, the assessment of the lip by patients also showed a statistically significant difference from the CMFS consultants (*p* = 0.002).

**Figure 4:**
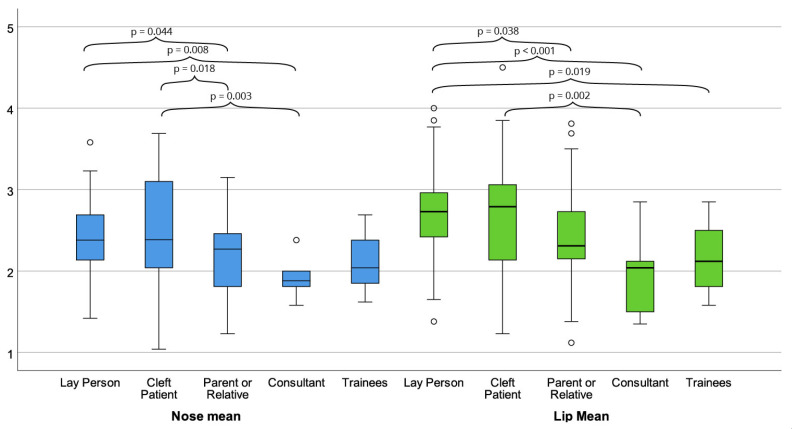
The graphic illustrates the statistically significant differences between the rater subgroups in their assessment of nasal appearance, shown in blue box plots, and labial aesthetics, depicted in green box plots (post hoc test).

Regarding the assessment of nasal appearance, the two most critical groups (laypersons and patients) showed a statistically significant difference from CMFS consultants and patient’s relatives. These findings strongly indicate a positive correlation between cleft experience levels and more favourable result ratings (Figure 4).

There were no significant gender differences in the assessments for either parameter according to the *T*-test (nose: *p* = 0.307; lip: *p* = 0.885).

### 3.4. Correlation Analysis

To investigate the relationship between averaged objective measurements and subjective assessments, the Pearson correlation coefficient (r) was employed.

**Figure 5:**
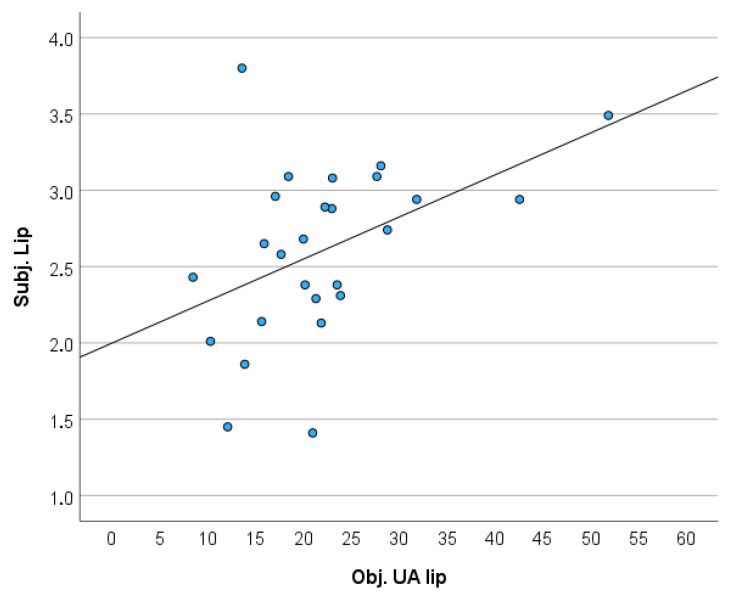
The scatter diagram displays a significant positive correlation between the objective measurement (UA lip) and the subjective assessment of the upper lip.

A significant positive correlation between the objective measurement of lip asymmetry (UA Lip) and subjective assessment was found (r = 0.455; *p* = 0.020) (Figure 5).

No significant correlation was observed between the subjective perception of the lip and objective measurement (CA Lip) (r = 0.381; *p* = 0.055), nor between the objective measurement of nose asymmetry (CAFR) and subjective evaluation (r = 0.371; *p* = 0.062). Upon dividing the raters into subgroups, most correlations were observed between the subjective assessment and the objective parameter UA Lip (Table 4).

## 4. Discussion

This study aims to assess postoperative outcomes on both objective and subjective levels and to identify differences in perception among rater subgroups with varying personal and professional backgrounds. In addition, the correlation between subjective assessments and objective measurements of the nasolabial region, obtained via SymNose software, was evaluated [9].

The principal finding of this study is the confirmation that subjective perception of the nasolabial region after unilateral cleft repair varies significantly based on the rater’s professional background and clinical experience with UCL(P) patients. The findings clearly demonstrate that laypersons and individuals affected by cleft condition rate the nasolabial region in UCL(P) patients more critically than CMFS consultants and residents in both nasal and lip assessment. The findings reported in the literature concerning this issue are inconclusive. In their systematic review, Zhu et al. analysed 11 studies that assessed the appearance of patients with facial deformities. In three studies, there was no significant difference between the assessments made by laypeople and professionals; in three studies, laypeople were more critical, in contrast to five studies in which professionals gave poorer assessments [15]. The pooling of the samples in this systematic review was not feasible due to different methodologies across the evaluated studies. In this study, we included 167 laypersons as raters. Comparable studies examining the soft tissue aesthetics of the nasolabial region in UCLP(P) patients did not include such a large number of layperson respondents. Chung et al. included 121 laypeople; however, the images assessed were of adult patients with treated cleft conditions, comparing results after Le-Fort osteotomy and late maxillary protraction [18].

Furthermore, at the time of writing this manuscript, no comparable study could be found in which the subjective assessment of the nasolabial region in patients after UCL(P) repair was examined by five different subgroups, including both patients affected by cleft condition and their relatives.

The systematic review conducted by Wang et al. [19] descriptively analysed 22 studies focusing on evaluations performed by professionals and patients affected by cleft clip and/or palate. Notably, nine studies reported that patients with cleft condition tended to rate postoperative outcomes more favourably than clinicians. Still, the overall findings of the review were indeterminate [19].

Although the majority of children and adults with cleft condition do not appear to suffer from major psychosocial problems, there was less satisfaction with facial appearance reported, as well as an increased tendency of depression and anxiety in these individuals [20].

In interpreting the results, it can be proposed that the heightened focus on the nasolabial region and inclination toward increased critical self-assessment among patients likely contributed to their more negative evaluations of nasolabial appearance. The studies focused on self-perception of the patients with cleft lip and/or palate showed that the majority of patients remain dissatisfied with their appearance after surgical therapy, and aesthetic deviations are more pronounced than functional impairments such as speech or nasal breathing difficulties [21,22]. It is well established that children with cleft condition face a higher risk of stigmatisation compared to peers without facial anomalies [23,24].

Interestingly, the ratings provided by laypersons were comparably unfavourable to those given by patients with cleft condition. It is important to consider that, for the layperson group, the images of the postoperative cleft nasolabial region likely represented their first substantial exposure to cleft appearance. The finding that CMFS consultants and residents provided the most lenient assessments may be attributed to their awareness of the complexity and technical demands of the surgical procedures required to achieve outcomes comparable to the nasolabial appearance of individuals without UCL(P).

Another key outcome of this study is the objective assessment of the nasolabial region in infants following primary cleft lip repair using the Fisher technique [6], measured with SymNose software. These data can be considered a solid basis for future comparisons with alternative surgical techniques.

Despite having undergone primary unilateral cleft lip repair, patients continue to demonstrate increased asymmetry compared to non-cleft controls, indicating that initial surgical correction does not fully restore facial symmetry to normative levels. Although nasolabial asymmetry is also present in healthy individuals, McKearney et al. [16] demonstrated that its extent is significantly lower compared to individuals with surgically corrected UCLP. In their study involving 22 UCLP patients, mean UA and CA lip were 20.2% (±11.7%) and 19.1% (±12.1%), respectively. In contrast, the control group (*n* = 22) demonstrated significantly lower values of 8.5% (±4.6%) and 9.9% (±4.9%) [16]. The level of asymmetry in their patient’s group aligns closely with the objective postoperative outcomes of this study.

In this study, lip asymmetry was measured at 22.09% (±9.45) for the UA lip and 22.43% (±9.2) for the CA lip. These values indicate a higher degree of asymmetry compared to the postoperative lip asymmetry reported by Merkl et al., who examined older patients following secondary lip revision surgery using the Fisher technique (*n* = 20), with mean asymmetry values of 18.27% for the UA lip and 18.77% for the CA lip [6,25]. These findings strongly support the notion that there remains room for aesthetic improvement when patients express a desire for further enhancement.

Regarding the nasal parameter CAFR, Merkl et al. [25] reported no significant improvement in nasal symmetry following lip revision surgery (preoperative 17.13% ± 5.91 vs. postoperative 17.78% ± 6.37; *p* = 0.642). In this cohort, the mean CAFR was higher at 19.94% ± 7.09. However, the differences were not statistically significant when compared using an independent two-sample *t*-test (*p* = 0.284). The results clearly show that neither primary cleft repair, including primary septorhinoplasty, nor secondary lip repair sufficiently corrects nasal asymmetry, underscoring the clinical importance of secondary septorhinoplasty in adulthood. Accordingly, comprehensive preoperative counselling is crucial to set realistic expectations.

Moreover, a moderate but statistically significant correlation was found between the objectively measured symmetry of the upper lip (UA lip) and its subjective aesthetic evaluation (r = 0.455, *p* = 0.020). This suggests that differences in lip symmetry are perceptible to evaluators and play a role in how postoperative aesthetics are judged.

In contrast, no significant correlation was observed between the subjective assessment of nasal appearance and the objective measurement of nasal asymmetry (CAFR), indicating that subjective evaluations do not consistently correspond with quantitative asymmetry metrics. It is important to consider that nasal symmetry, as captured by the CAFR, reflects only one dimension of nasal morphology. The results imply that nasal aesthetic especially should be considered more holistically, with additional soft tissue and contour factors not fully captured by linear measurements. It may be suggested that 3D scans would be more suitable for the objective assessment of the nasal region. Due to improved availability and standardisation, as well as the validation of the SymNose evaluation programme, 2D images were used in this study. However, future studies addressing this topic should also verify the results using 3D images.

## 5. Limitations

Firstly, the distribution of respondents across the subjective assessment cohorts was not balanced. However, it is not desirable to limit the number of respondents solely for the purpose of achieving a balanced ratio of subgroups. Secondly, conducting multiple subjective assessments with the same raters may have minimised the risk of bias. However, this was not feasible due to the large number of respondents. Thirdly, the study population of this investigation consisted exclusively of Caucasian patients; therefore, no generalisability for all populations can be proclaimed. Fourthly, the study did not include parent-proxy reported outcome measures and put the focus exclusively on the results of one surgical primary cleft lip repair technique. However, proxy-PROMs and the comparison of surgical techniques would have gone beyond the scope of this paper.

Finally, the data collected provides a solid basis for future comparative studies on objective and subjective outcomes in the nasolabial area. However, the attractiveness of a face is not defined solely by this specific area. Numerous other features, which were not considered in this study and are also significantly influenced by cultural and personal factors, ultimately determine the individual perception of the aesthetics of a face.

## 6. Conclusions

The results of this study demonstrate that aesthetics are in the eye of the beholder, particularly in relation to cleft lip. Laypeople and patients with cleft lip +/− palate are subjectively more critical in their assessment of the repaired nasolabial areas than individuals who are more familiar with these conditions. This is an important consideration, as the patients’ everyday environment typically consists of laypeople. Secondary procedures should therefore be actively discussed with affected patients and offered to them in order to prevent psychosocial complaints and improve quality of life.

## Figures and Tables

**Table 1:** Intrarater and interrater reliability coefficient for rater 1 and rater 2 concerning the objective assessment.

	CAFR	CA Lip	UA Lip
Rater 1: Exam 1 vs. 2	ICC = 0.964	ICC = 0.922	ICC = 0.914
Rater 2: Exam 1 vs. 2	ICC = 0.939	ICC = 0.925	ICC = 0.889
Rater 1 vs. Rater 2	ICC = 0.967	ICC = 0.967	ICC = 0.938

**Table 2:** Characteristics of the subgroups concerning sex and age.

Assessor Details	*n*	Male	Female	Age Mean	Age SD
Total	255	111	144	32.3	11.34
Layperson	167	70	97	27	10.52
Cleft patient	32	17	15	17.8	11.08
Cleft patient’s relative	38	11	27	39.9	9.65
CMFS resident	9	7	2	30.1	2.37
CMFS consultant	9	6	3	46.9	11.77

**Table 3:** The subjective assessment results using the Cleft Aesthetic Rating Scale (CARS).

		*n*	Min	Max	Mean	SD	95% CI	*p*-Value
Nose	Layperson	167	1.42	3.58	2.40	0.38	[2.09–2.72]	*p* < 0.001
	Cleft patient	32	1.04	3.69	2.51	0.68	[2.25–2.76]	
	Parent’s relative	38	1.23	3.15	2.18	0.47	[1.92–2.45]	
	CMFS resident	9	1.62	2.69	2.12	0.38	[1.83–2.40]	
	CMFS consultant	9	1.58	2.38	1.90	0.23	[1.59–2.21]	
Lip	Layperson	167	1.38	4.00	2.69	0.44	[2.44–2.94]	*p* < 0.001
	Cleft patient	32	1.23	4.50	2.68	0.70	[2.46–2.90]	
	Parent or relative	38	1.12	3.81	2.43	0.61	[2.23–2.64]	
	CMFS resident	9	1.58	2.85	2.16	0.44	[1.95–2.36]	
	CMFS consultant	9	1.35	2.85	1.95	0.54	[1.74–2.16]	

**Table 4:** Detailed correlations between the objective measurement and subjective assessment after dividing the study participants into subgroups.

Subjective Analysis	CAFR		CA Lip		UA Lip	
Nose layperson	r = 0.381	*p* = 0.055	-	-	-	-
Nose cleft patient	r = 0.346	*p* = 0.083	-	-	-	-
Nose pat. relative	r = 0.325	*p* = 0.105	-	-	-	-
Nose consultant	r = 0.401	*p* = 0.042	-	-	-	-
Nose resident	r = 0.304	*p* = 0.131	-	-	-	-
Lip layperson	-	-	r = 0.335	*p* = 0.094	r = 0.420	*p* = 0.033
Lip cleft patient	-	-	r = 0.392	*p* = 0.048	r = 0.449	*p* = 0.021
Lip pat. relative	-	-	r = 0.481	*p* = 0.013	r = 0.539	*p* = 0.004
Lip consultant	-	-	r = 0.284	*p* = 0.159	r = 0.285	*p* = 0.158
Lip resident	-	-	r = 0.606	*p* = 0.001	r = 0.585	*p* = 0.002

## Data Availability

The data that support the findings of this study are available from the corresponding author upon reasonable request.
